# Doença de Kawasaki Incompleta Diagnosticada Apenas com Febre Prolongada: Relato de Dois Casos e Revisão da Literatura

**DOI:** 10.36660/abc.20230163

**Published:** 2024-06-26

**Authors:** Hikmet Kiztanir, Ayse Sulu, Pelin Kosger, Tugcem Akin, Birsen Ucar

**Affiliations:** 1 Recep Tayyip Erdogan Universitesi Egitim ve Arastirma Hastanesi Rize Turquia Recep Tayyip Erdogan Universitesi Egitim ve Arastirma Hastanesi - Pediatric Cardiology, Rize – Turquia; 2 Eskisehir Osmangazi University Faculty of Medicine Eskisehir Turquia Eskisehir Osmangazi University Faculty of Medicine - Pediatric Cardiology, Eskisehir – Turquia

**Keywords:** Doença de Kawasaki Incompleta Síndrome de Linfonodos Mucocutaneous, Aneurisma Coronário, Febre de Causa Desconhecida

## Introdução

A doença de Kawasaki (DK) é uma vasculite febril aguda de causa desconhecida. A DK incompleta (atípica) foi definida para pacientes que não atendem aos critérios diagnósticos característicos clássicos. A incidência de desenvolvimento de aneurisma de artéria coronária é maior em pacientes com DK incompleta devido a atrasos no diagnóstico e tratamento. Na literatura, os pacientes que não atendem aos critérios diagnósticos mas são diagnosticados apenas com febre são muito raros. Aqui, apresentamos dois casos de DK que foram diagnosticados com aneurisma de artéria coronária e achados laboratoriais de suporte sem sinais característicos além de febre alta. O primeiro caso foi de uma menina de 3 anos que foi diagnosticada com DK após um exame ecocardiográfico no 14º dia de febre revelar um aneurisma de artéria coronária.^1-3^O segundo caso foi diagnosticado no 8º dia de febre devido ao envolvimento das artérias coronárias sem outros achados clássicos de Kawasaki. Ambos os pacientes receberam antibioterapia com diagnósticos diferentes, como gastroenterite e infecção do trato urinário, antes do diagnóstico de DK. Ambos os casos foram diagnosticados antes da pandemia de COVID-19.^4^ Portanto, a doença inflamatória multissistêmica em crianças (MIS-C) não foi considerada. Nosso objetivo foi enfatizar a importância de considerar a DK em crianças com febre inexplicável e altos reagentes de fase aguda, mesmo na ausência de critérios diagnósticos característicos, e a necessidade de controles seriados para avaliar pacientes suspeitos com ecocardiografia e outros achados laboratoriais de suporte

### Caso 1

**Figura 1:**
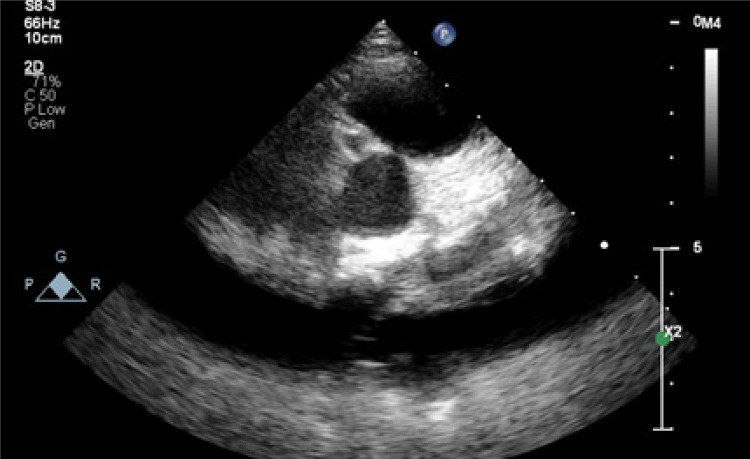
– Ecocardiografia transtorácica demonstrou dilatações saculares no meio da artéria coronária direita.

Uma menina de três anos foi internada no Departamento de Infectologia Pediátrica do nosso hospital com febre de até 39°C que persistiu por 9 dias. Nos acompanhamentos hospitalares anteriores, soube-se que ela apresentava fortes dores abdominais inferiores com febre, altos reagentes de fase aguda, leucocitose e piúria na análise de urina, e estava recebendo tratamentos com ceftriaxona e amicacina com diagnóstico preliminar de infecção do trato urinário. Devido à presença de reagentes de fase aguda elevados e à ausência de sinais de DK clássica, foram realizadas as consultas e exames necessários para excluir patologias como abscessos intra-abdominais e colecistite no Departamento de Infectologia Pediátrica. Então, no 4º dia de internação, foi consultada pelo nosso Serviço de Cardiologia Pediátrica para avaliação de DK. Sua história médica e familiar não era digna de nota. Ao exame físico, seu peso corporal era de 17 kg (percentil 75-90) e sua altura era de 98 cm (percentil 25-50). Nenhum sinal de KD clássico foi encontrado. No hemograma, não houve outra característica além de leucocitose em dominância de neutrófilos, proteína total: 6,3 g/dL, albumina sérica: 2,8 g/dL, proteína C reativa (PCR): 7,3 mg/dL (faixa normal: 0- 0,8 mg/dL) e velocidade de hemossedimentação (VHS):86 mm/h. A ecocardiografia transtorácica (ETT) revelou aneurismas saculares de tamanho médio nas principais artérias coronárias direita e esquerda (ACD e ACE) pelo ETT [4,3 x 4 mm na ACD (escore z: 6,66) e 5,2 x 4,9 mm na ACE (escore z: 6.65)] (Figura 1). Com o diagnóstico de DK, o paciente recebeu 2 gr/kg de imunoglobulina intravenosa (IVIG) e uma dose anti-inflamatória de ácido acetilsalicílico. Observou-se descamação nas pontas dos dedos no dia seguinte. A paciente não apresentou febre após terapia com IVIG e os reagentes de fase aguda regrediram no acompanhamento da paciente. No 9º dia de tratamento, o nível de PCR foi reduzido para 0,6 mg/dL, enquanto a contagem de plaquetas e o nível de albumina sérica aumentaram para 923.000/mm3 e 4,4 g/dL, respectivamente. Na ecocardiografia de controle realizada 2 semanas depois, foi detectada redução no tamanho dos aneurismas das artérias coronárias [2,4 x 1,8 mm na ACD (escore z: 1,23) e 4,1 x 3,8 mm na ACE (escore z: 3,97)]. Na segunda semana de acompanhamento, a dose de tratamento com ácido acetilsalicílico foi reduzida para uma dose antiagregante. A angiografia coronária seletiva realizada no 8º mês de seguimento revelou trajeto coronário normal e diâmetros sem aspecto compatível com aneurisma.

### Caso 2

**Figura 2:**
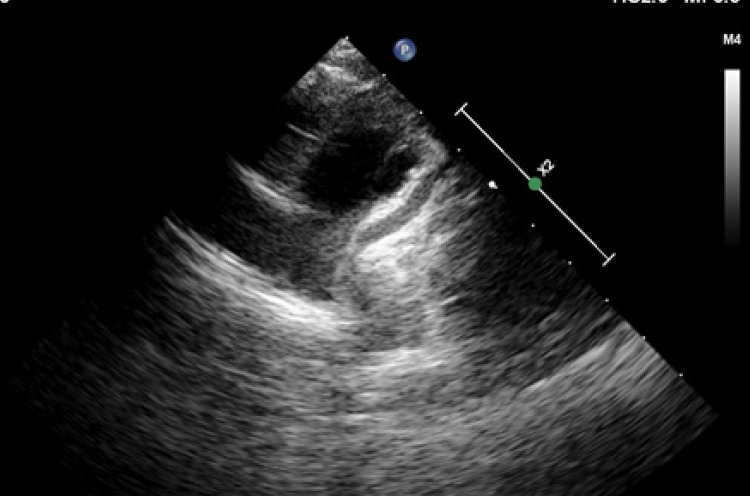
– Ecocardiografia transtorácica demonstrou aparente ectasia (3,5 mm; escore z 5,58) na artéria coronária descendente anterior esquerda.

Uma menina de seis anos foi internada em nosso hospital devido a febre persistente e dor de cabeça. Soube-se que treze dias antes da internação em nosso hospital, ela havia sido internada em outro ambulatório com diagnóstico de gastroenterite por apresentar febre que chegava a 39,5°C associada a diarreia e dor abdominal e foi tratada com ceftriaxona e metronidazol intravenosos. Como a febre persistiu por mais de 5 dias (8 dias) e os níveis de reagentes de fase aguda estavam elevados, ela foi avaliada com ETT com diagnóstico provisório de DK. A ecocardiografia revelou dilatação fusiforme na artéria coronária descendente anterior esquerda (ADE). Após receber 2 gr/kg de IVIG e dose anti-inflamatória de ácido acetilsalicílico, a febre regrediu e ela recebeu alta do outro hospital um dia depois. Ela foi internada em nosso hospital no segundo dia após receber alta por causa de febre recorrente superior a 38°C. Soube-se que o paciente não apresentava sinais de DK clássica durante esses períodos. A história médica e familiar do paciente era normal. No exame físico; a altura era de 105 cm (percentil 25-50) e o peso corporal era de 17 kg (percentil 10-25). Achados clássicos de KD não foram observados. Em exames laboratoriais; o hemograma não apresentava alterações, exceto trombocitose (contagem de plaquetas 508.000/mm^3^), níveis séricos de proteína total 8,1 g/dL, albumina 3,6 g/dL, procalcitonina 0,099 ng/mL (valor normal: 0-0,5 ng/mL), PCR 34,4 mg/dL (valor normal: 0-5 mg/dL) e VHS 93 mm/h. Não foi detectada piúria no exame de urina. Não houve crescimento nas culturas de sangue e urina, as bactérias da flora normal cresceram nas culturas de fezes. O eletrocardiograma estava normal. No ETT, havia ectasia aparente (3,5 mm) na ADE (escore z: 5,58) (Figura 2), e o tronco da coronária esquerda (TCE) estava dilatado para 3,5 mm (escore z: 2,61). Continuando a terapia oral com ácido acetilsalicílico na dose anti-inflamatória de 100 mg/kg, foi administrada uma segunda sessão de terapia IVIG na dose de 2 g/kg. A febre não recorreu após a segunda terapia com IVIG e ela recebeu alta no sétimo dia de internação. No décimo dia de internação (no acompanhamento ambulatorial), os níveis de PCR e VHS diminuíram para 2,8 mg/dL e 7 mm/h, respectivamente, e a contagem de plaquetas aumentou para 621.000/mm^3^. No acompanhamento ambulatorial no décimo quarto dia de internação, embora não tenha havido alteração significativa no tamanho da ACE (3,6 mm, escore z 5,94), foi detectada redução no tamanho do TCE (2,6 mm, escore z: 0,38). Na segunda semana de acompanhamento, a dose de tratamento com ácido acetilsalicílico foi reduzida para uma dose antiagregante. Com diminuição gradativa do tamanho do aneurisma no período de seguimento, a avaliação ecocardiográfica no nono mês de seguimento revelou regressão completa das dilatações coronarianas. A cineangiocoronariografia realizada no décimo segundo mês também foi normal.

## Discussão

O diagnóstico de DK incompleta ou atípica é feito pela ecocardiografia e pela presença de achados laboratoriais de apoio nos pacientes com febre persistente há pelo menos 5 dias associada a dois ou três critérios clínicos. Foi relatado que 12% dos pacientes com DK apresentavam uma forma incompleta. Na diretriz de 2017 da American Heart Association, recomenda-se o início do tratamento em crianças com febre persistente por pelo menos 5 dias e dois ou três critérios clínicos compatíveis ou em lactentes com febre há pelo menos sete dias sem outros critérios clínicos que apresentem PCR elevada ( ≥3 mg/dL) e VHS (≥40 mm/h) em caso de presença de pelo menos três dos achados laboratoriais de suporte (anemia, contagem de plaquetas ≥450.000/mm^3^ após o 7º dia de febre, albumina ≤3 g /dL, nível elevado de aspartato transaminase (AST), contagem de leucócitos ≥15.000/mm^3^, ≥10 leucócitos/CGA na urinálise) ou um achado compatível na avaliação ecocardiográfica.^1^ Em nossos casos, aqueles que tiveram febre persistente por mais de 5 dias e achados laboratoriais de inflamação sistêmica, aneurismas de artérias coronárias e ectasias foram detectados por ecocardiografia. No entanto, nenhum dos sintomas clássicos da DK esteve presente em nossos casos, exceto a descamação nas pontas dos dedos que ocorreu no período subagudo após o diagnóstico no caso 1. O desenvolvimento de aneurismas de artéria coronária geralmente começa após o 10º dia de doença. Para pacientes diagnosticados após o décimo dia, recomenda-se a administração de tratamento com IVIG em caso de presença de febre ou anomalia da artéria coronária com persistência de níveis elevados de VHS e PCR.^1-3^ Devido ao encaminhamento um tanto tardio do caso 1 para nós no final do 13º dia de doença, foram detectados aneurismas de artérias coronárias durante a fase subaguda da doença, e a paciente recebeu IGIV devido à persistência de febre e sinais inflamatórios sistêmicos. Após a fase aguda da doença, a terapia com ácido acetilsalicílico é reduzida a uma dose antiagregante para fornecer terapia antitrombótica. Quando há um aneurisma da artéria coronária, recomenda-se medicação com baixas doses de ácido acetilsalicílico por toda a vida, mesmo que os achados da artéria coronária tenham voltado ao normal. Em pacientes com aneurismas moderados ou grandes, recomenda-se combinar ácido acetilsalicílico com heparina de baixo peso molecular ou varfarina.^1^ Se a febre persistir após 36 horas de tratamento com IVIG, uma segunda dose de infusão de IVIG, uma combinação de corticosteroides e IVIG , ou tratamento com infliximabe é recomendado, considerando KD resistente.^1,4,5^No caso 2, a febre e os níveis elevados de reagentes de fase aguda persistiram após a infusão inicial de IVIG, mas o paciente se beneficiou do segundo tratamento com IVIG. Na presença de febre persistente e elevação dos reagentes de fase aguda que não possam ser explicadas por qualquer outro motivo, deve-se considerar a DK incompleta. Ressaltou-se que a maioria desses pacientes com evolução atípica tem menos de um ano e apresentavam eritema na cicatriz da vacina BCG, inquietação e diarreia.^6-8^ Da mesma forma, ambos os nossos pacientes foram acompanhados com diagnósticos diferentes devido a queixas de dor abdominal e diarreia. Dor de cabeça foi uma queixa concomitante em nosso segundo caso. Não foi observado eritema na cicatriz da BCG em nossos pacientes. No entanto, as idades dos pacientes na literatura cujos diagnósticos foram baseados apenas na presença de febre sem acompanhamento de achados característicos são variáveis. Os achados de nossos casos, juntamente com os casos relatados apenas com febre na literatura, estão resumidos na Tabela 1. Yeom et al.,^9^ compararam em seus estudos os achados de seis pacientes com meningite asséptica por DK e os pacientes com meningite causada por infecção por enterococos. Como os achados clínicos e laboratoriais dos pacientes de Kawasaki com meningite asséptica não foram apresentados individualmente, os detalhes são desconhecidos, mas foi relatado que dois desses pacientes inicialmente não apresentavam nenhum dos achados característicos da DK.^9^ Apresentamos dois casos de DK que foram diagnosticados devido apenas a febre persistente, para enfatizar que a DK deve ser considerada na presença de febre persistente inexplicável e achados laboratoriais sugestivos de inflamação sistêmica, sem achados clássicos de DK. Nestes pacientes, os achados laboratoriais de apoio devem ser analisados e as artérias coronárias devem ser avaliadas por ecocardiografia e, se a febre persistir, repetidas e acompanhadas de perto. Além disso, são necessárias mais pesquisas inovadoras para identificar marcadores imunológicos e celulares que possam ser testados nos estágios iniciais da doença e orientar o manejo.

**Tabela 1:** – Resumo dos nossos casos de doença de Kawasaki diagnosticados apenas com febre e casos semelhantes na literatura

	Caso 1	Caso 2	Vignesh (7)	Uchida (10)	Ozdemir (11)	Tapa (12)	Yilmazer (13)	Cabral (14)	Yeom (9) Caso 1	Yeom (9) Caso 2
Idade	3 anos	6 anos	5 anos	20 meses	2,5 meses	7 meses	8 meses	3 meses	3 meses	3 meses
Gênero	F	F	M	M	M	M	F	F	M	M
Duração de febre (dia)	13	11	17	12	12	7	19	22	10	5
Critérios diagnósticos característicos	-	-	-	-	-	-	-	Erupção cutânea, descamação (no 22º dia)	-	-
Sintomas adicionais	Dor abdominal	Dor abdominal, diarreia, dor de cabeça	Irritabilidade	Irritabilidade	Nenhum	Irritabilidade	Nenhum	Diarreia	?	?
Hemoglobina (g/dL)	10	11	9	?	8.3	11,5	9,2	8,9	?	?
WBC (/mm^3^)	17.300	13.000	16.750	12.700	16.700	11.500	12.100	10.900	?*	?*
Plaquetas (/mm^3^)	430.000	508.000	518.000	626.000	710.000	350.000	988.000	891.000	?*	?*
Albumina (g/dL)	2.8	3.6	2	?	?	3.4	?	2.6	?*	?*
ALT (U/L)	11	10	?	Normal	17	?	?	Alto	Normal	Normal
Piúria	+	-	?	?	?	?	?	?	?*	?*
Proteína C-reativa (mg/dl)	7.3	34,4	26,8	131	8.3	48	22,3	6,7	?*	?*
VHS (mm/hora)	96	93	97	?	80	42	125	122	?*	?*
Descobertas adicionais	-	-	-	-	-	-	-	Pleocitose	Pleocitose	Pleocitose
Ecocardiografia	Escore z ACD: 6,66 (4,3 x 4 mm) Escore z do TCE: 6,65 (5,2 x 4,9 mm)	escore z do TCE 2,6 (3,5 mm) Escore z ACE: 5,58 (3,5 mm)	Escore z da ACE: 16 Escore z ACD: 10	TCE: 3,7 mm LCX: 4,3mm ACD: 4,3mm	Escore z ACD: 7,25 Escore z do TCE: 7,6	ACE: 4,8 mm	TCE: 6 mm	ACD: 3,4 mm ACE: 4,2 mm	Envolvimento coronariano (+)	Normal
Tratamento	IVIG, aspirina	IVIG (2 doses), aspirina	IVIG + infliximabe, HBPM, aspirina	IVIG, aspirina	IVIG, aspirina	IVIG, aspirina	IVIG, aspirina	IVIG, aspirina	?	?

*Os resultados laboratoriais não foram relatados individualmente neste estudo. Dados de um total de 6 pacientes [mediana (limites inferior-superior)]: leucócitos: 12.340 (6.500-23.930)/mm^3^, plaquetas: 420.000 (246.000-518.000)/mm^3^, albumina: 3,7 (3,1-4,4) mg/dL , ALT: 20 (16-23) UI/L, piúria em 50% dos pacientes, PCR: 46,5 (37-115) mg/dL. F: feminino; M: masculino; IVIG: imunoglobulina intravenosa; VHS: velocidade de hemossedimentação, leucócitos: glóbulos brancos; ACD: artéria coronária direita; TCE: tronco da coronária esquerda; ACE: artéria descendente anterior esquerda; HBPM: baixo peso molecular heparina.
