# Supplementary material for: Doença de Kawasaki Incompleta Diagnosticada Apenas com Febre Prolongada: Relato de Dois Casos e Revisão da Literatura
Source: Arq Bras Cardiol. 2024 Jun 26;121(6):e20230163. [Article in Portuguese] doi: 10.36660/abc.20230163 (PMC11216336; doi:10.36660/abc.20230163)
Supplement: Translation

# Translation: Incomplete Kawasaki Disease Diagnosed only with Prolonged Fever: Report of Two Cases and Review of the Literature

**DOI:** 10.36660/abc.20230163i

**Authors:** Hikmet Kiztanir, Ayse Sulu, Pelin Kosger, Tugcem Akin, Birsen Ucar

**Affiliations:** 1 Turkey Recep Tayyip Erdogan Universitesi Egitim ve Arastirma Hastanesi - Pediatric Cardiology, Rize – Turkey; 2 Turkey Eskisehir Osmangazi University Faculty of Medicine - Pediatric Cardiology, Eskisehir – Turkey

**Keywords:** Incomplete Kawasaki Disease, Mucocutaneous Lymph Node Syndrome

## Introduction

Kawasaki disease (KD) is an acute febrile vasculitis of unknown cause. Incomplete (atypical) KD has been defined for patients who do not meet the classical characteristic diagnostic criteria. The incidence of coronary artery aneurysm development is higher in patients with incomplete KD due to delays in diagnosis and treatment. In the literature, patients who do not meet the diagnostic criteria but are diagnosed solely with fever are very rare. Here, we present two cases of KD who were diagnosed with coronary artery aneurysm and supportive laboratory findings with no characteristic signs other than high fever. The first case was a 3-year-old girl who was diagnosed with KD after an echocardiographic examination on the 14th day of the fever revealed a coronary artery aneurysm.^1-3^ The second case was diagnosed on the 8th day of the fever because of the involvement of the coronary arteries without other classic Kawasaki findings. Both patients had been given antibiotherapy with different diagnoses such as gastroenteritis and urinary tract infection before the diagnosis of KD. Both cases were diagnosed before the COVID-19 pandemic.^4^ Therefore, multisystem inflammatory disease in children (MIS-C) was not considered. We aimed to emphasize the importance of considering KD in children with unexplained fever and high acute phase reactants, even in the absence of characteristic diagnostic criteria, and the need for serial controls to evaluate suspected patients with echocardiography and other supportive laboratory findings

### Case 1

A three-year-old girl was admitted to the Pediatric Infectious Diseases Department of our hospital with a fever of up to 39°C that persisted for 9 days. In the prior hospital follow-ups, it was learned that she had severe lower abdominal pain with fever, high acute phase reactants, leukocytosis, and pyuria in the urine analysis, and she has been receiving ceftriaxone and amikacin treatments with a preliminary diagnosis of urinary tract infection. Because of the presence of high acute phase reactants and no signs of classic KD, the necessary consultations and examinations were performed to exclude pathologies such as intra-abdominal abscesses and cholecystitis in the Pediatric Infectious Diseases Department. Then, on the 4th day of her hospitalization, she was consulted by our Department of Pediatric Cardiology to be evaluated for KD. Her medical and family history were unremarkable. On physical examination, her body weight was 17 kg (75-90 percentile) and her height was 98 cm (25-50 percentile). No signs of classic KD were found. In the blood count, there was no feature other than leukocytosis in neutrophil dominance, total protein: 6.3 g/dL, serum albumin: 2.8 g/dL, C-reactive protein (CRP): 7.3 mg/dL (normal range: 0-0.8 mg/dL), and erythrocyte sedimentation rate (ESR):86 mm/hr. Transthoracic echocardiography (TTE) revealed medium-sized saccular aneurysms in the right and left main coronary arteries (RCA and LCA) by TTE [4.3 x 4 mm in RCA (z score: 6.66) and 5.2 x 4.9 mm in LCA (z score:6.65)] (Figure 1). With the diagnosis of KD, the patient was administered 2 gr/kg intravenous immunoglobulin (IVIG) and an anti-inflammatory dose of acetylsalicylic acid. Desquamation was observed on the fingertips on the next day. The patient did not have a fever after IVIG therapy and acute phase reactants regressed in the follow-up of the patient. On the 9th day of the treatment, CRP level was reduced to 0.6 mg/dL while platelet count and serum albumin level increased to 923,000/mm3 and 4.4 g/dL, respectively. In the control echocardiography performed 2 weeks later, a reduction in the size of the coronary artery aneurysms was detected [2.4 x 1.8 mm in RCA (z score: 1.23) and 4.1 x 3.8 mm in LCA (z score:3.97)]. On the second week of follow-up, the dose of acetylsalicylic acid treatment was decreased to an antiaggregant dose. Selective coronary angiography performed at the 8th month of the follow-up revealed normal coronary artery course and diameters with no appearance compatible with an aneurysm.

**Figure 1:** – Transthoracic echocardiography demonstrated saccular dilations in the middle of the right coronary artery.

### Case 2

A six-year-old girl was admitted to our hospital due to persistent fever and headache. It was learned that thirteen days before admission to our hospital, she had been hospitalized at another clinic with the diagnosis of gastroenteritis because she had a fever reaching 39.5°C associated with diarrhea and abdominal pain and was treated with intravenous ceftriaxone and metronidazole. Because her fever persisted longer than 5 days (8 days) and acute phase reactant levels were elevated, she had been evaluated with TTE with the provisional diagnosis of KD. Echocardiography revealed a fusiform dilatation in the left anterior descending coronary artery (LAD). After receiving 2 gr/kg of IVIG and an anti-inflammatory dose of acetylsalicylic acid, her fever had regressed and she had been discharged from the other hospital one day later. She was admitted to our hospital on the second day after being discharged because of recurrent fever exceeding 38°C. It was learned that the patient had no signs of classical KD during these periods. The patient’s medical and family history was unremarkable. In physical examination; height was 105 cm (25-50 percentile) and body weight was 17 kg (10-25 percentile). Classical KD findings were not observed. In laboratory tests; the blood count was unremarkable except for thrombocytosis (platelet count 508,000/mm3), serum levels of total protein 8.1 g/dL, albumin 3.6 g/dL, procalcitonin 0.099 ng/mL (normal value: 0-0.5 ng/mL), CRP 34.4 mg/dL (normal value:0-5 mg/dL), and ESR 93 mm/hr. Pyuria was not detected in the urinalysis. There was no growth in blood and urine cultures, normal flora bacteria grew in stool culture. The electrocardiogram was normal. In TTE, there was an apparent ectasia (3.5 mm) in LAD (z score: 5.58) (Figure 2), and the left main coronary artery (LMCA) was dilated to 3.5 mm (z score:2.61). Continuing oral acetylsalicylic acid therapy at the anti-inflammatory dose of 100 mg/kg, a second session of IVIG therapy at a dose of 2 g/kg was given. Her fever did not recur following the second IVIG therapy and she was discharged on the seventh day of admission. On the tenth day of admission (at out-patient follow-up), CRP and ESR levels were decreased to 2.8 mg/dL and 7 mm/hr, respectively and platelet count increased to 621,000/mm^3^. In the outpatient follow-up on the fourteenth day of hospital admission, although there was no significant change in LAD size (3.6 mm, z score 5.94), a reduction in LMCA size (2.6 mm, z score: 0.38) was detected. On the second week of follow-up, the dose of acetylsalicylic acid treatment was decreased to an antiaggregant dose. With a gradual decrease of aneurysmal size in the follow-up period, the echocardiographic evaluation on the ninth month of follow-up revealed a complete regression of the coronary dilations. Coronary angiography performed on the twelfth month was also normal.

**Figure 2:** – Transthoracic echocardiography demonstrated an apparent ectasia (3.5 mm; z score 5.58) in the left anterior descending coronary artery.

## Discussion

The diagnosis of incomplete or atypical KD is made by echocardiography and the presence of the supporting laboratory findings in the patients with at least 5 days persisting of fever associated with two or three clinical criteria. It has been reported that 12% of KD patients had an incomplete form. In the 2017 guideline of the American Heart Association, beginning treatment is recommended in children with fever persisting for at least 5 days and two or three compatible clinical criteria or in infants with fever for at least seven days without other clinical criteria who have high CRP (≥3 mg/dL) and ESR (≥40 mm/hr) in case of the presence of at least three of the supportive laboratory findings (anemia, platelet count of ≥450,000/mm^3^ after the 7th day of fever, albumin ≤3 g/dL, elevated aspartate transaminase (AST) level, leucocyte count of ≥15,000/mm^3^, ≥10 leucocyte/hpf in urinalysis) or a compatible finding in echocardiographic evaluation.^1^ In our cases, those who had a fever persisting for more than 5 days and laboratory findings of systemic inflammation, coronary artery aneurysms, and ectasias were detected by echocardiography. However, none of the classic KD symptoms were present in our cases, except desquamation at the fingertips occurred in the subacute period after diagnosis in case 1. The development of coronary artery aneurysms often starts after the 10th day of the disease. For patients who were diagnosed after the tenth day, it is recommended to administer IVIG treatment in case of the presence of fever or coronary artery abnormality with the persistence of elevated ESR and CRP levels.^1-3^ Due to case 1’s late referral to us somewhat late on the 13th day of the disease, coronary artery aneurysms were detected during the subacute phase of the disease, and the patient was given IVIG because of the persistence of fever and systemic inflammatory signs. After the acute phase of the disease, acetylsalicylic acid therapy is reduced to an antiaggregant dose to provide antithrombotic therapy. When there is a coronary artery aneurysm, lifelong low-dose acetylsalicylic acid medication is advised, even if the coronary artery findings have returned to normal. In patients with moderate or large-sized aneurysms, it is recommended to combine acetylsalicylic acid with low molecular-weight heparin or warfarin.^1^ If fever persists after 36th hours of IVIG treatment, a second dose of IVIG infusion, a combination of corticosteroids and IVIG, or infliximab treatment is recommended, considering resistant KD.^1,4,5^ In case 2, the fever and elevated acute phase reactant levels persisted after the initial IVIG infusion, but the patient benefited from the second IVIG treatment. In the presence of persistent fever and elevation in acute phase reactants that cannot be explained by any other reason, incomplete KD should be considered. It was highlighted that most of these patients with atypical course are under one year old and had erythema in BCG vaccine scar, restlessness, and diarrhea.^6-8^ Similarly, both of our patients had been followed up with different diagnoses due to complaints of abdominal pain and diarrhea. Headache was an accompanying complaint in our second case. Erythema at the BCG scar was not observed in our patients. However, the ages of the patients in the literature whose diagnoses were based only on the presence of fever without accompanying characteristic findings are variable. The findings of our cases along with the cases reported with only fever in the literature are summarized in Table 1. Yeom et al.^9^ compared the findings of six patients with aseptic meningitis due to KD and the patients with meningitis caused by enterococcus infection in their study. Because the clinical and laboratory findings of the Kawasaki patients with aseptic meningitis were not individually presented, the details are unknown, but it was reported that two of these patients initially had presented none of the characteristic findings of KD.^9^ We presented two cases of KD who were diagnosed due to only persistent fever, to emphasize that KD should be considered in the presence of unexplained persistent fever and laboratory findings suggestive of systemic inflammation, without classical findings of KD. In these patients, supporting laboratory findings should be analyzed and coronary arteries should be evaluated by echocardiography, and if fever persists, repeated and followed-up closely. Additionally, further innovative research is very much needed to identify immunological and cellular markers that can be tested in the early stages of the disease and guide management.

**Table 1:** – A summary of our Kawasaki disease cases diagnosed only with fever and similar cases in the literature

	Case 1	Case 2	Vignesh (7)	Uchida (10)	Ozdemir (11)	Thapa (12)	Yılmazer (13)	Cabral (14)	Yeom (9) Case 1	Yeom (9) Case 2
Age	3 year	6 year	5 year	20 month	2.5 month	7 month	8 month	3 month	3 month	3 month
Gender	F	F	M	M	M	M	F	F	M	M
Duration of fever (day)	13	11	17	12	12	7	19	22	10	5
Characteristic diagnostic criteria	-	-	-	-	-	-	-	Rash, peeling (at 22^nd^ day)	-	-
Additional symptoms	Abdominal pain	Abdominal pain, diarrhea, headache	Irritability	Irritability	None	Irritability	None	Diarrhea	?	?
Hemoglobin (g/dL)	10	11	9	?	8.3	11.5	9.2	8.9	?	?
WBC (/mm^3^)	17,300	13,000	16,750	12,700	16,700	11,500	12,100	10,900	?*	?*
Platelet (/mm^3^)	430,000	508,000	518,000	626,000	710,000	350,000	988,000	891,000	?*	?*
Albumin (g/dL)	2.8	3.6	2	?	?	3.4	?	2.6	?*	?*
ALT (U/L)	11	10	?	Normal	17	?	?	High	Normal	Normal
Pyuria	+	-	?	?	?	?	?	?	?*	?*
C- reactive protein (mg/dl)	7.3	34.4	26.8	131	8.3	48	22.3	6.7	?*	?*
ESR (mm/hour)	96	93	97	?	80	42	125	122	?*	?*
Additional findings	-	-	-	-	-	-	-	Pleocytosis	Pleocytosis	Pleocytosis
Echocardiography	RCA z score: 6.66 (4.3 x 4 mm) LMCA z score: 6.65 (5.2 x 4.9 mm)	LMCA z score 2.6 (3.5 mm) LAD z score: 5.58 (3.5 mm)	LAD z score: 16 RCA z score: 10	LMCA: 3.7 mm LCX: 4.3 mm RCA: 4.3 mm	RCA z score: 7.25 LMCA z score: 7.6	LAD: 4.8 mm	LMCA: 6 mm	RCA: 3.4 mm LCA: 4.2 mm	Coronary involvement (+)	Normal
Treatment	IVIG, aspirin	IVIG (2 doses), aspirin	IVIG + infliximab, LMWH, aspirin	IVIG, aspirin	IVIG, aspirin	IVIG, aspirin	IVIG, aspirin	IVIG, aspirin	?	?

*Laboratory findings were not reported individually in this study. Data of a total of 6 patients [median (lower-upper limits)]: WBC: 12,340 (6,500- 23,930)/mm^3^, platelet: 420,000 (246,000- 518,000)/mm^3^, albumin: 3.7 ( 3.1- 4.4) mg/dL, ALT: 20 (16-23) IU/L, pyuria in 50% of the patients, CRP: 46.5 (37-115) mg/dL. F: female; M: male; IVIG: intravenous immunoglobulin; ESR: erythrocyte sedimentation rate; WBC: white blood cell; RCA: right coronary artery; LMCA: left main coronary artery; LAD: left anterior descending artery; LMWH: low molecular weight heparin.
